# Forest Age Rivals Climate to Explain Reproductive Allocation Patterns in Forest Ecosystems Globally

**DOI:** 10.1111/ele.70191

**Published:** 2025-08-25

**Authors:** Rachel E. Ward, Huanyuan Zhang‐Zheng, Kate Abernethy, Stephen Adu‐Bredu, Luzmilla Arroyo, Andrew Bailey, Jos Barlow, Erika Berenguer, Liana Chesini‐Rossi, Percival Cho, Cecilia A. L. Dahlsjö, Eder Carvalho das Neves, Bianca de Oliveira Sales, William Farfan‐Rios, Joice Nunes Ferreira, Renata Freitag, Cécile Girardin, Walter Huaraca Huasco, Carlos A. Joly, Yadvinder Malhi, Beatriz Marimon, Ben Hur Marimon Junior, Alexandra C. Morel, Helene C. Muller‐Landau, Karine da Silva Peixoto, Simone Reis, Terhi Riutta, Norma Salinas, Marina Seixas, Miles R. Silman, Lara M. Kueppers

**Affiliations:** ^1^ Energy and Resources Group University of California, Berkeley Berkeley California USA; ^2^ Environmental Change Institute, School of Geography and the Environment University of Oxford Oxford UK; ^3^ Universidad Nacional de la Amazonia Peruana Iquitos Peru; ^4^ Instituto de Investigaciones de la Amazonía Peruana Iquitos Peru; ^5^ Forestry Research Institute of Ghana, Council for Scientific and Industrial Research Kumasi Ghana; ^6^ Museo de Historia Natural Noel Kempff Mercado Santa Cruz de la Sierra Bolivia; ^7^ Leverhulme Centre for Nature Recovery The Oxford University Centre for the Environment Oxford UK; ^8^ Lancaster Environment Centre Lancaster University Lancaster UK; ^9^ Rio de Janeiro Botanical Garden Research Institute Rio de Janeiro Brazil; ^10^ Science for Sustainability Ltd Belmopan Belize; ^11^ Programa de Pós‐Graduação Em Ecologia e Conservação (PPGEC) Universidade do Estado de Mato Grosso (UNEMAT) Nova Xavantina‐MT Brazil; ^12^ Laboratório de Ecologia Vegetal (LABEV) Universidade do Estado de Mato Grosso (UNEMAT) Nova Xavantina‐MT Brazil; ^13^ Department of Biology Wake Forest University Winston‐Salem North Carolina USA; ^14^ Sabin Center for Environment and Sustainability Wake Forest University Winston‐Salem North Carolina USA; ^15^ Embrapa Amazônia Oriental Belém Brazil; ^16^ Faculdade de Educação e Ciéncias Sociais Aplicadas (FAECS) Universidade do Estado de Mato Grosso (UNEMAT) Campus de Juara‐MT Brazil; ^17^ Nature‐Based Insights & Nature‐Based Solutions Initiative University of Oxford Oxford UK; ^18^ Department of Plant Biology Biology Institute, University of Campinas, UNICAMP Campinas‐SP Brazil; ^19^ Division of Energy, Environment and Society University of Dundee Dundee UK; ^20^ Smithsonian Tropical Research Institute Panamá Panama; ^21^ Centro de Ciências Biológicas e da Natureza, Universidade Federal Do Acre Rio Branco‐AC Brazil; ^22^ Department of Science, Chemistry Section Institute for Nature Research Earth and Energy, Pontifical Catholic University of Peru Lima Peru; ^23^ Climate and Ecosystem Sciences Division Lawrence Berkeley National Laboratory Berkeley California USA

**Keywords:** climate, ecosystem modelling, forest age, forest ecosystems, forest regeneration, reproductive allocation, soil fertility

## Abstract

Forest allocation of net primary productivity (NPP) to reproduction (carbon required for flowers, fruits, and seeds) is poorly quantified globally, despite its critical role in forest regeneration and a well‐supported trade‐off with allocation to growth. Here, we present the first global synthesis of a biometric proxy for forest reproductive allocation (RA) across environmental and stand age gradients from a compiled dataset of 824 observations across 393 sites. We find that ecosystem‐scale RA increases ~60% from boreal to tropical forests. Climate shows important non‐linear relationships with RA, but is not the sole predictor. Forest age effects are comparable to climate in magnitude (MAT: ß = 0.24, *p* = 0.021; old growth forest: ß = 0.22, *p* < 0.001), while metrics of soil fertility show small but significant relationships with RA (soil pH: ß = 0.07, *p* = 0.001; soil N: ß = −0.07, *p* = 0.001). These results provide strong evidence that ecosystem‐scale RA is mediated by climate, forest age, and soil conditions, and is not a globally fixed fraction of positive NPP as assumed by most vegetation and ecosystem models. Our dataset and findings can be used by modellers to improve predictions of forest regeneration and carbon cycling.

## Graphical abstract

Forest allocation of net primary productivity (NPP) to reproduction is poorly quantified globally, despite its critical role in forest regeneration and a well‐supported trade‐off with allocation to growth. We present the first global synthesis of forest reproductive allocation (RA) using a comprehensive dataset of 824 observations across 393 sites worldwide. We find that ecosystem‐scale RA increases ~60% from boreal to tropical forests and is mediated by climate, forest age, and soil fertility, with forest age effects rivalling those of climate. These findings challenge the current assumption in vegetation and ecosystem models that RA is a fixed fraction of NPP and provide empirical data to improve predictions of forest regeneration and carbon cycling.

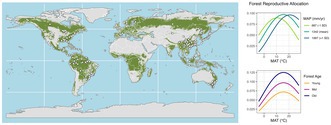

## 1 Introduction

The allocation of net primary productivity (NPP) to reproduction in forests refers to the carbon allocated to fruits, flowers, seeds, cones, and other reproductive structures. Reproductive allocation (RA) is the first step of tree regeneration, and although fundamental to forest persistence and trophic interactions, remains one of the least studied carbon fluxes in forest ecosystems (Wenk and Falster 2015). Understanding global patterns in RA is critical for predicting forests' capacity for regeneration, resilience to disturbance, and long‐term carbon dynamics. While other major carbon fluxes are well documented across forests globally (e.g., Anderson‐Teixeira et al. 2021; Luyssaert et al. 2007; Yang et al. 2021), patterns in RA have been difficult to characterise due to measurement challenges and historical research priorities focused on wood production and carbon sequestration (Pugh et al. 2020). Understanding RA is particularly critical given its well‐supported trade‐off with allocation to growth, which influences both individual fitness and ecosystem‐level carbon cycling (Hanbury‐Brown et al. 2022; Wenk et al. 2018; but see also Bazzaz et al. (1987) for discussion of trade‐offs between reproduction, growth, and defence). Substantial variation in reproductive investment across forests globally would also have large implications for our understanding of the web of biotic interactions supported by reproductive materials.

Climate shapes forest function through its influence on resource availability and environmental constraints, determining both plant's available energy and allocation patterns (Anderson‐Teixeira et al. 2021; Reich et al. 2014). At the ecosystem scale, there is some evidence physiological constraints interact with life history strategies to shape reproductive patterns across global climate gradients. Resource‐rich environments favour species with rapid growth and shorter lifespans, which tend to mature and reproduce earlier, while resource‐limited environments select for species with slower growth and extended longevity that typically delay reproduction, prioritising future over current reproductive investment (Bazzaz et al. 1987; Locosselli et al. 2020; Obeso 2002; Pugliese and Kozlowski 1990). This pattern extends to reproductive frequency: boreal and temperate species typically reproduce at annual or longer intervals, while tropical species may reproduce continuously (Kelly and Sork 2002; Morellato et al. 2018; Sakai 2001). Dominant plant types, resulting from environmental filtering as well as evolutionary history, may also shape patterns; needle‐leaved conifers dominating boreal and some temperate ecosystems typically exhibit slower growth and greater longevity compared to broadleaf species (Locosselli et al. 2020). Although reproductive strategies are diverse (Salguero‐Gómez et al. 2016) and RA remains poorly quantified for long‐lived species (Wenk and Falster 2015), large‐scale differences are expected to drive systematic variation in forest ecosystem carbon allocation to reproduction, increasing from boreal to tropical forest ecosystems.

While biome‐level patterns reflect broad biogeographic constraints, variation in RA may be more accurately explained by underlying temperature gradients, which control NPP through multiple pathways (Michaletz et al. 2014). Warmer environments with longer growing seasons may allow sustained simultaneous growth and reproduction, while colder environments with shorter growing seasons may require greater carbon allocation to functions supporting survival (e.g., root growth, Reich et al. 2014). Recent empirical evidence strongly supports temperature as a driver of reproductive investment, suggesting a 250‐fold increase in community seed production across a combined temperature‐moisture gradient that corresponds with only a threefold increase in NPP (Journé et al. 2022). However, this prior analysis focuses solely on seed production. Whether temperature drives a similar increase in total RA—encompassing flowers, fruits, and other reproductive structures in addition to seeds—remains unclear.

Moisture availability may constrain RA through different mechanisms at both extremes of the precipitation gradient. Under moisture limitation, reduced carbon fixation combined with increased allocation to survival‐critical hydraulic architecture (e.g., sapwood and root tissue) likely reduces carbon available for reproduction (Flexas et al. 2006; Obeso 2002; Poorter et al. 2012). However, long‐term studies present contrasting evidence: some find that moisture stress limits RA primarily through reduced seed development rather than changes in flowering effort (Pérez‐Ramos et al. 2010), while others suggest that at the ecosystem scale, flowering and fruiting may show an immediate decrease in response to drought, but recover to higher than pre‐drought levels, perhaps due to changes in species composition (Rowland et al. 2018). At the other extreme, excessive precipitation can constrain reproduction either through physiological stress from anoxic soil conditions (Kozlowski 1986) or reduce carbon fixation when persistent cloud cover limits radiation (Detto et al. 2018). While regional studies have documented precipitation effects on reproductive output, the relationship between precipitation and RA—and its interaction with temperature—has not been quantified at global scales.

Reproductive tissues are nutrient dense compared to vegetative structures (Reekie and Bazzaz 1987), suggesting soil fertility may limit RA (Obeso 2002). Limited evidence from neotropical forests supports a link between nutrient availability and reproductive investment (Chave et al. 2010; Fortier and Wright 2021; Kaspari et al. 2008; Minor and Kobe 2019). However, nutrient addition experiments reporting reproductive and total net primary productivity at the ecosystem scale find no significant effects on RA (Adamek et al. 2009; Alvarez‐Clare et al. 2013). Understanding these relationships is further complicated by soil physical and chemical properties that mediate nutrient availability to plants, including soil texture, pH, and cation exchange capacity (Binkley and Vitousek 1989; Silver et al. 2000).

Forest age may act as a control on ecosystem‐scale RA through multiple pathways, including tree size distributions, tree species composition, and tree size at reproductive maturity. While individual trees increase or begin carbon allocation to reproduction after reaching reproductive maturity—typically at half of their maximum adult stature (Minor and Kobe 2019; Visser et al. 2016; although see Journé et al. 2024) – stand‐level RA may exhibit non‐linear patterns due to successional dynamics, in which young stands exhibit elevated RA due to reduced light competition enabling reproduction at smaller sizes (Wright et al. 2005), and dominance by fast‐reproducing early successional species (Bazzaz 1979). As stands develop, intensifying competition may delay reproduction while community composition shifts towards species that prioritise vertical growth before reproducing, potentially reducing stand‐level RA during mid‐succession, but increasing in late‐succession as a greater proportion of trees reach reproductive maturity (Falster et al. 2017).

Here, we present the first global synthesis of a biometric proxy for forest RA. We hypothesise that RA will: (1) increase from boreal to temperate to tropical forests, with lower values in needleleaf compared to broadleaf forests; (2) show a positive relationship with temperature that plateaus or declines at high temperatures due to heat stress, interacting with moisture; (3) display a unimodal response to moisture, declining in extremely dry and wet conditions; (4) increase with soil fertility, particularly N and P availability; and (5) vary non‐linearly with stand age. We anticipate that climate variables will show the strongest relationship with RA, reflecting both greater variation in climate variables at the global scale and empirical evidence that temperature and precipitation are dominant drivers of seed production globally (Journé et al. 2022; Moles et al. 2009). We expect stand age effects to show intermediate strength, as forest development influences tree maturity and ecosystem‐level resource allocation patterns. Soil variables (texture, CEC, pH) are expected to have relatively weaker associations with RA, as their effects may be modulated by the stronger influence of climate (Simpson et al. 2016).

## 2 Methods

### 2.1 Estimation of Ecosystem‐Level Reproductive Allocation

**FIGURE 1:**
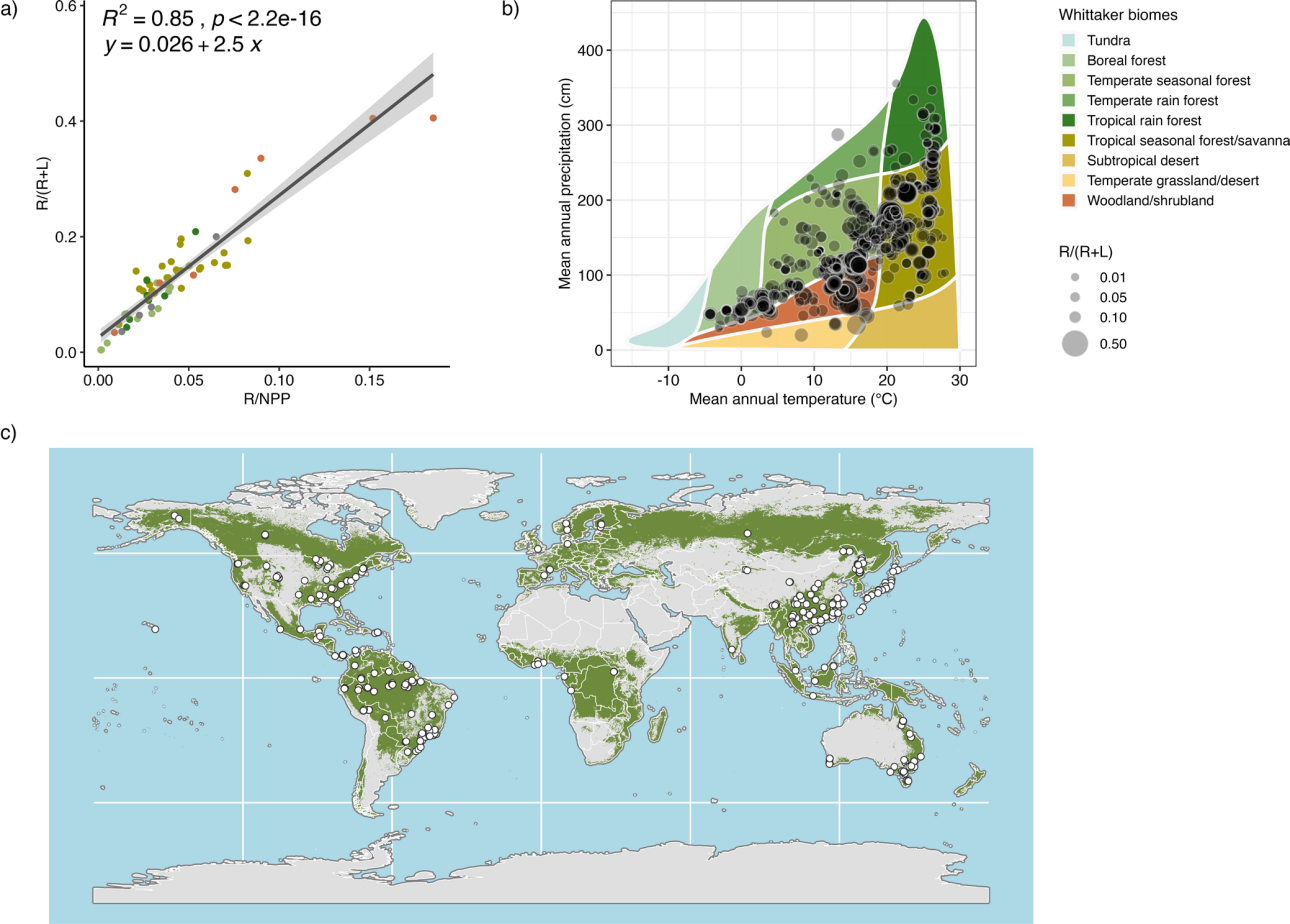
(a) R/NPP is strongly correlated with a litterfall proxy for RA, R/(R + L), at forested sites (*n* = 66, R = reproductive litterfall, L = leaf litterfall, NPP = net primary productivity, all fluxes in Mg ha^−1^yr ^−1^), adapted from Hanbury‐Brown et al. (2022); point colours indicate Whittaker biomes as shown in (b). (b) Observations of R/(R + L) are distributed across Whittaker biome space. Point size indicates annual average R/(R + L), overlapping points appear darker. (c) Observations of R/(R + L) are distributed across the forested (green) continents.

To assess ecosystem‐level RA, we would ideally compare observations of the fraction of net primary productivity (NPP) dedicated to reproductive tissues (R/NPP) across forests globally. However, field measurements of total NPP are rare in the literature, and measurement protocols vary among studies, undermining inter‐comparison (Clark et al. 2001; Luyssaert et al. 2007). For this reason, we use a proxy, R/(R + L), constructed solely from litterfall observations (R and L signify annual reproductive and leaf litterfall fluxes respectively, in Mg ha^−1^ year^−1^), which are much more widely available globally. Our previous work identified 66 sites where reproductive litterfall is reported as a distinct component of NPP and found a striking correlation between R/NPP and R/L (*R* ^2^ = 0.87; Hanbury‐Brown et al. 2022). We use a variant of this proxy, R/(R+ L), which is preferable for analysis because it is bounded between zero and one, and find a strong correlation between R/(R + L) and R/NPP (Figure 1a; *R* ^2^ = 0.85, see Figure S1 for correlation between log‐transformed variables, *R* ^2^ = 0.94), supporting its use as a proxy for RA across a wide range of forest sites where biometric NPP estimates are unavailable. Strong correlation is driven in part by correlation between denominators (Figure S2). We also tested total litterfall as a denominator (R/Total litterfall) because of its reported correlation with NPP in previous work (Aragão et al. 2009; Bray and Gorham 1964; Girardin et al. 2010; Malhi et al. 2011). However, we find that (R + L) is more strongly correlated with NPP than total litterfall, likely because of inconsistent protocols for the inclusion of non‐leaf and non‐reproductive materials across sites globally (Clark et al. 2001).

### 2.2 Data Collection

We conducted a comprehensive search for peer‐reviewed publications, data papers, and databases reporting annual reproductive and leaf litterfall fluxes from forested ecosystems, excluding savanna. We searched Google Scholar, Web of Science, and SciELO using predefined search terms (litterfall OR litter fall OR litter‐fall OR litter*) AND (fruit OR flower OR seed OR cone OR reproductive*) AND (leaf OR leaves OR foliar OR foliage OR needle) AND (tree OR forest OR ecosystem). From the initial ~300,000 publications, we used article titles and abstracts to exclude studies focused on individual species, experimental manipulations, and plantation forests, as the latter exhibit artificially uniform stand age biassing ecosystem‐level RA estimates. We retained only references reporting ecosystem‐level reproductive and leaf litterfall components separately from total litterfall for a minimum of one year of sampling. Our search included literature in English, Spanish, and Portuguese, and drew on previous litterfall synthesis efforts encompassing the primary literature from Russia, China, and Japan (Holland et al. 2015; Jia et al. 2016; Martinelli et al. 2017; Neumann et al. 2021; Suzuki et al. 2012), and data published by ecological monitoring networks (e.g., NEON). In addition, we obtained unpublished litterfall flux and soil characteristic data from the GEM network (Malhi et al. 2021). Duplicate sites were identified and removed.

For each site, we recorded geographic coordinates, climate variables, litterfall fluxes, sampling protocols, forest characteristics (dominant leaf morphology, information pertaining to forest age and successional stage, and disturbance history) and soil properties (% sand, %silt, %clay), nitrogen (N), total phosphorus (P), cation exchange capacity (CEC), and pH where available (see Supporting Information). Due to variable sampling needs across biomes and inconsistent protocol reporting in databases, we did not exclude sites based on litterfall trap specifications or collection frequency. When necessary, we computed annual mean litterfall fluxes (standardised to units Mg ha^−1^ year^−1^) from publicly available temporal datasets or extracted values from figures using WebPlotDigitizer (Rohatgi 2021). All litterfall fluxes reported in terms of carbon were standardised to dry biomass units using site‐specific biomass to carbon ratios when reported, or 0.49 (Ma et al. 2018). The final dataset comprised 824 observations (1583 observation‐years) from 393 unique geographic locations spanning the forested continents (Figure 1c, Table S1), with sampling durations ranging from 1 to 24 years (Figure S3).

### 2.3 Climate Data

To characterise sites by their long‐term mean climatic conditions, we extracted estimates from WorldClim 2.1. For each unique set of geographic coordinates, we extracted 30‐year (1970–2000) mean annual temperature (MAT) and mean annual precipitation (MAP) at 30‐arcsec (about 1 km) spatial resolution (Fick and Hijmans 2017; Table S2). We used mean MAT and MAP estimates to characterise sites in the Whittaker biome space using the R package ‘plotbiomes’ (Ștefan and Levin 2018, Figure 1b). We then classified forest sites as tropical, temperate, or boreal using latitude (absolute degrees latitude > 50° = boreal, 50°–25° = temperate, < 25° = tropical) and Whittaker biome classifications. We checked for agreement between latitude and Whittaker‐based biome classifications to identify edge cases. When latitude and Whittaker biome classifications disagreed (61 sites), we consulted the reference publication to determine biome.

### 2.4 Soil Characteristics

Soil characteristics of interest were reported for only 67 sites. In order to test relationships between RA and soil characteristics across our full range of data, we extracted predictions of soil pH, N, CEC, and texture (%sand, %silt, %clay) from SoilGrids250 (Hengl et al. 2017), and computed the depth‐weighted average to 30 cm (Table S2). Estimates to 30 cm depth are consistent with reported soil properties from GEM/RAINFOR sites (Quesada et al. 2010) and were also computed for NEON ecological monitoring network sites (NEON 2024), which constituted the majority of sites reporting soil characteristics in our dataset. For the analysis of soil texture, we use a soil texture index defined as the additive log ratio, log (% sand/% clay) (Aitchison 1982; Lark and Bishop 2007; Poggio et al. 2021). Total phosphorus is not predicted by SoilGrids, and therefore was not included as a predictor in our main model. However, on‐site total phosphorus was reported for 34 sites. On‐site soil characteristics were used for robustness checks (see Methods S1).

### 2.5 Stand Age

Forest age (years) and qualitative information about successional stage were reported for 500 and 614 sites, respectively. We mapped successional stage to age categories (“early successional” to young, “mid‐successional/secondary” to mid, and “late successional/primary/old growth” to old), and using 290 sites where both metrics were available, we established biome‐specific age thresholds to categorise forests into three age classes (young, mid, and old; Table S3). To maximise site inclusion while accounting for biome‐specific differences in forest growth rates and successional dynamics, we differentiated age classes by biome for tropical (< 20, 20–60, > 60 years), temperate (< 40, 40–100, > 100 years), and boreal (< 50, 50–120, > 120 years) forests (Locosselli et al. 2020). When both metrics were available, we prioritised age‐based classification.

### 2.6 Statistical Analyses

We analysed patterns in reproductive allocation (R/(R + L)) using two approaches. First, we tested differences across forest biomes and plant types using one‐way ANOVA with Tukey's HSD post hoc comparisons. Data were Box‐Cox transformed to meet assumptions of normality and homoscedasticity (Box and Cox 1964, Figure S4).

To examine relationships between RA and climate, soil, and stand age, we fit linear mixed effects models using the ‘nlme’ package (Pinheiro et al. 2023). To better understand how predictors influence the individual components of the RA proxy R/(R + L), we developed parallel models for Box‐Cox transformed reproductive (R) and leaf (L) litterfall fluxes. All numerical covariates were standardised and centred for effect size comparison (Table S2).

Models tested for both linear and non‐linear climate effects by including quadratic terms for mean annual temperature and precipitation (MAT^2^, MAP^2^) and their interaction (MAT × MAP). For all response variables, we analysed model residuals to evaluate whether the fitted quadratic models accurately captured the shape of the relationships throughout the range of the data. To account for site‐specific variation and sampling effort, models incorporated site‐level random effects and observations weighted by the square root of the sampling duration (years). This weighting approach accounts for increased precision from long‐term measurements without allowing them to dominate the analysis. For all models, we used backwards selection to retain significant predictors (*p* < 0.05) while preserving interaction terms that substantially influenced main effects (see Methods S2, Table S4, and Figures S5–S11 for full model specifications and diagnostics).

Finally, we tested the sensitivity of our model to alternate weighting and forest stand age classification schemes, for which coefficient estimates proved highly robust (see Tables S5–S7). For the final analysis, we employed the weighting scheme described above, and the forest age classification scheme described in the 'Stand Age' section. While this approach may accentuate age‐related effects, it aligns with our goal of examining how RA patterns shift across broad stages of forest development.

## 3 Results

**FIGURE 2:**
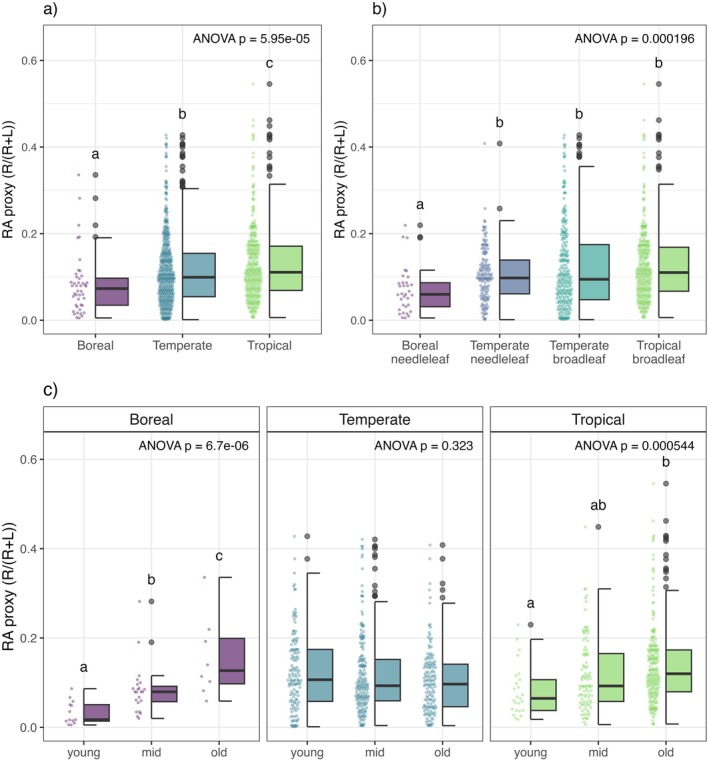
Boxplots of RA proxy R/(R + L) across (a) biomes (*n* = 824) and (b) plant types defined by biome and dominant leaf morphology (*n* = 716; sites with undetermined or mixed dominant leaf morphology excluded) and (c) biome and forest age classification (*n* = 824). Panels show results from ANOVA and Tukey's HSD post hoc comparisons (indicated with letters) on Box‐Cox transformed data (raw data shown in plot). Boxplots show median, quartiles, and 1.5*IQR whiskers.

Annual RA varied by two orders of magnitude in our dataset, ranging from 0.0014 to 0.5456. Across biomes, we found that RA exhibited a ~60% increase from boreal forests (mean = 0.082) to tropical forests (mean = 0.129), with intermediate values observed in temperate forests (mean = 0.116). Biome‐level differences were statistically significant (Figure 2a, Tables S8 and S9), confirming our expectation of significant variation in reproductive allocation across forests globally. Consistent with the expectation of highly variable reproductive material fluxes, standard deviations were high across all biomes (SD = 0.070, 0.084, and 0.087 for boreal, temperate, and tropical forests, respectively), likely reflecting a combination of sampling error and true biological variation among sites. Although the rank order of RA over plant types aligned with our expectations, only boreal needleleaf RA was statistically different from other plant types, providing only weak support for our expectation that plant type moderates biome‐level patterns (Figure 2b, Tables S10 and S11).

### 3.1 Climate

**FIGURE 3:**
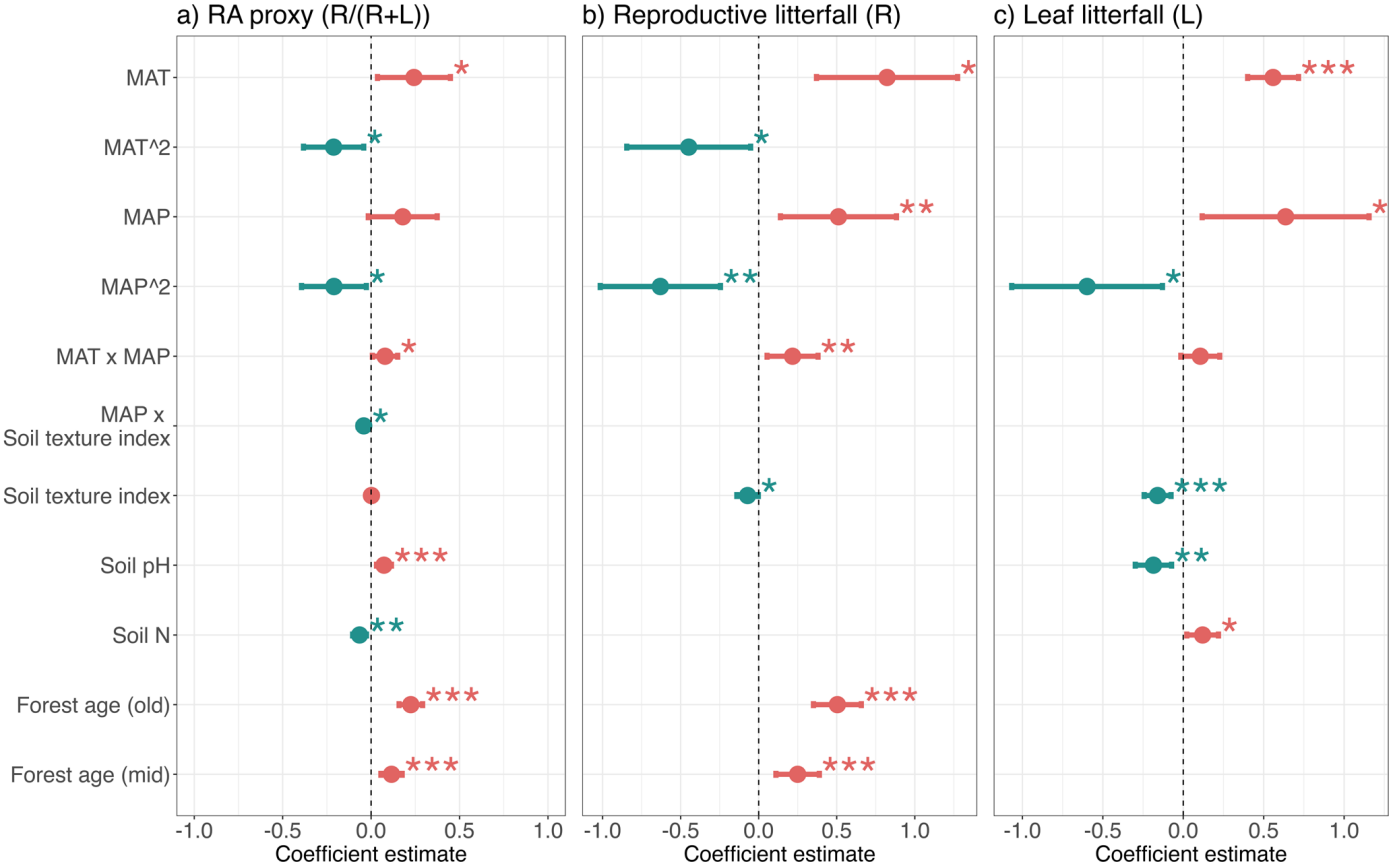
Standardised coefficient estimates (points) and 95% confidence intervals (lines) from final models predicting a) RA proxy (R/(R+ L)), (b) Reproductive litterfall (R) and (c) Leaf litterfall (L). MAT = mean annual temperature (°C), MAP = mean annual precipitation (mm/year), Forest age (young) is the reference level for the categorical variable Forest age, asterisks indicate statistical significance (*p < 0.05, **p < 0.01, ***p < 0.001).

**FIGURE 4:**
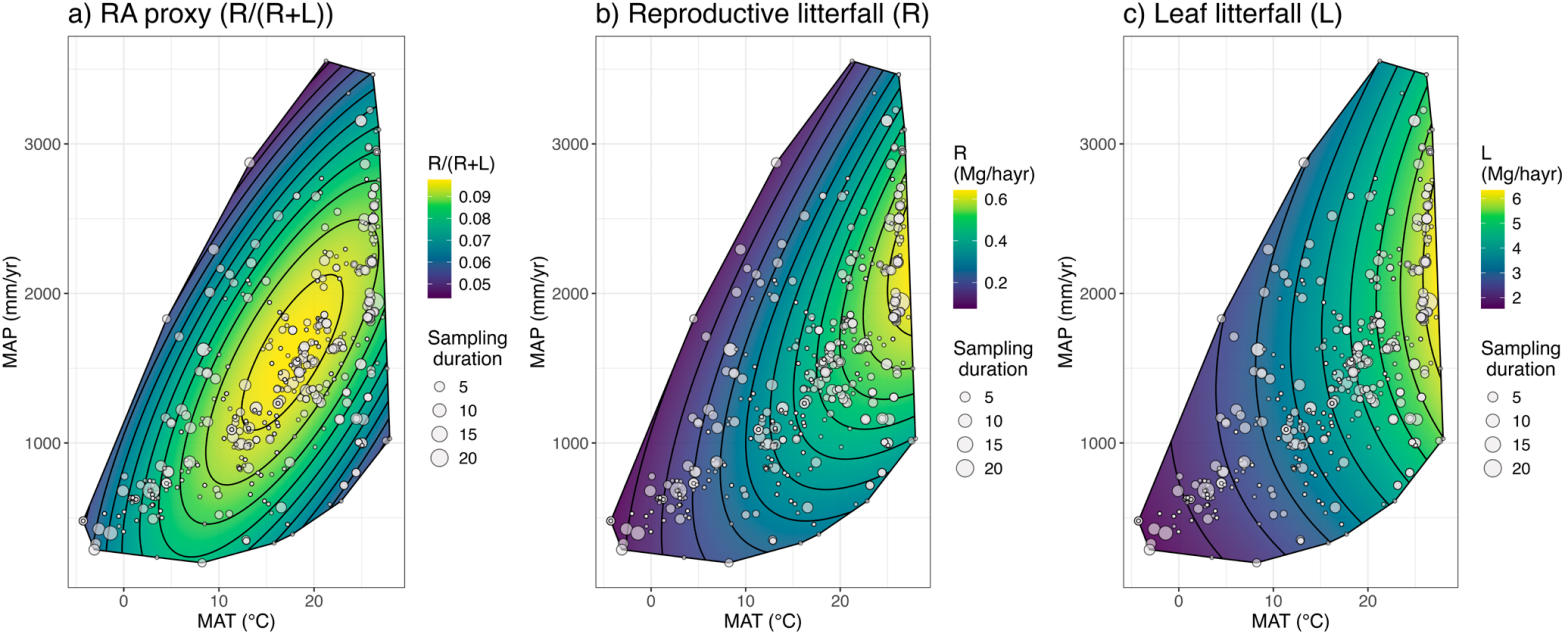
Predicted (a) RA proxy (R/ (R + L)), (b) Reproductive litterfall (R), and (c) Leaf litterfall (L) from our final model (Figure 3, Table 1) in mid‐aged forests with mean soil texture, mean soil pH, and mean soil N, as a function of mean annual temperature (MAT) and mean annual precipitation (MAP). Convex hulls are defined by observations, plotted in grey (point size reflects sampling duration in years).

Consistent with observed biome‐level differences, models show significant relationships between RA and climate predictors (Figure 3, Table 1), with a peak in RA at approximately 18°C and 1500 mm annual precipitation (Figure 4). Supporting our hypothesis that temperature stress may influence reproductive allocation across the global climatic temperature range, we found evidence for a unimodal relationship between mean annual temperature and RA in the final linear mixed effects model (Figure 3a, Table 1).

**TABLE 1:** Final model coefficients, confidence intervals (CI) and *p*‐values for response variables RA proxy (R/(*R* + L)), reproductive litterfall (R) and leaf litterfall (L). Prior to model fitting, all response variables were Box‐Cox transformed, and all numerical variables were scaled and centred (MAT = mean annual temperature, MAP = mean annual precipitation).

	RA proxy (R/(R +L))			Reproductive litterfall (R)			Leaf litterfall (L)		
Predictors	Estimates	CI	*p*	Estimates	CI	*p*	Estimates	CI	*p*
(Intercept)	−1.76	−1.84 to −1.69	**< 0.001**	−1.34	−1.52 to −1.16	**< 0.001**	1.75	1.64 to 1.87	**< 0.001**
MAT	0.24	0.04 to 0.45	**0.021**	0.82	0.37 to 1.27	**< 0.001**	0.56	0.40 to 0.72	**< 0.001**
MAT2	−0.21	−0.38 to −0.04	**0.015**	−0.45	−0.85 to −0.05	**0.027**			
MAP	0.18	−0.02 to 0.37	0.072	0.51	0.14 to 0.88	**0.007**	0.64	0.12 to 1.16	**0.016**
MAP2	−0.21	−0.39 to −0.03	**0.025**	−0.63	−1.01 to −0.24	**0.001**	−0.60	−1.07 to −0.13	**0.013**
Soil pH	0.07	0.03 to 0.12	**0.001**				−0.19	−0.30 to −0.07	**0.001**
Soil N	−0.07	−0.10 to −0.03	**0.001**				0.12	0.02 to 0.22	**0.017**
Soil texture index	0.00	−0.03 to 0.03	0.911	−0.07	−0.14 to −0.00	**0.043**	−0.16	−0.24 to −0.08	**< 0.001**
Forest age [mid]	0.12	0.06 to 0.18	**< 0.001**	0.25	0.11 to 0.39	**< 0.001**			
Forest age [old]	0.22	0.16 to 0.29	**< 0.001**	0.50	0.35 to 0.66	**< 0.001**			
MAT × MAP	0.08	0.01 to 0.15	**0.032**	0.22	0.05 to 0.38	**0.009**	0.11	−0.02 to 0.23	0.086
MAP × Soil texture index	−0.04	−0.07 to −0.01	**0.011**						
**Random effects**									
σ^2^	0.04			0.20			0.18		
τ_00_	0.04 _site_			0.27 _site_			0.39 _site_		
ICC	0.53			0.57			0.69		
N	393 _site_			393 _site_			393 _site_		
Observations	824			824			824		
Marginal *R* ^2^/Conditional *R* ^2^	0.135/0.592			0.283/0.695			0.500/0.843		
AIC	183.613			1576.918			1616.229		

*Note:* Bold *p*‐values indicate statistical significance (*p* < 0.05).

The increase in RA with precipitation was modestly enhanced at higher temperatures. We also found evidence for a unimodal relationship with MAP, suggesting potential moisture constraints on RA, which in extremely moist conditions may reflect high cloudiness. The effect of MAP was further mediated by soil texture, with smaller precipitation‐associated increases in RA in sites with coarser soil texture (Figure 3a, Table 1).

Climate effects were more pronounced for reproductive litterfall flux alone, with stronger unimodal effects of temperature and precipitation than observed for RA, while leaf litterfall flux showed only a positive linear relationship with mean annual temperature and a weakly significant concave down relationship with mean annual precipitation (Figure 3b,c, Table 1).

### 3.2 Soil Fertility

Hypotheses related to soil fertility effects on RA were partially supported. Given that lower soil acidity promotes fertility through increased nutrient solubility and microbial activity (Binkley and Vitousek 1989), we expected a positive association between pH and allocation to reproduction if nutrients are limiting. Consistent with this expectation, we found a positive effect of pH on RA (Figure 3a, Table 1). However, we did not find evidence for a positive effect of soil N, and instead found a weak negative effect on RA (Figure 3a, Table 1). We found no support for a positive effect of cation exchange capacity (CEC), and it was removed from final models to avoid overfitting (Table S4). We cannot rule out the possibility that the significant interaction between MAP and soil texture reflects an allocation response to differences in soil nutrient supply associated with soil texture, in addition to texture‐mediated effects of water availability (Figure 3a, Table 1). To test whether relationships between RA and soil conditions are equally relevant across biomes, we ran versions of our global RA model within temperate and tropical biomes (we did not run a boreal model due to lack of data, *n* = 44). However, we did not find evidence for stronger soil effects within each biome compared to the global model encompassing all biomes (data not shown).

Reproductive litterfall showed a modest negative relationship with soil texture index, but no other significant relationships with soil variables (Figure 3b, Table 1). In contrast, leaf litterfall exhibited multiple significant relationships with soil properties, including negative associations with both soil pH and soil texture index, and a positive relationship with soil N (Figure 3c, Table 1). These patterns suggest higher leaf production in more acidic, fine‐textured, and nitrogen‐rich soils, and more pronounced edaphic effects for leaf than reproductive productivity.

Robustness checks using on‐site soil measurements partially supported these findings, although only soil texture remained a significant soil predictor of leaf litterfall (Table S14). On‐site soil measurements also provided an opportunity to test hypotheses related to soil total P, though analysis was limited by sample size (*n* = 34). We observed weak positive correlations with response variables, strongest for RA (*R* ^2^ = 0.069, *p* = 0.133), but none reached statistical significance (Figure S12).

### 3.3 Forest Age

**FIGURE 5:**
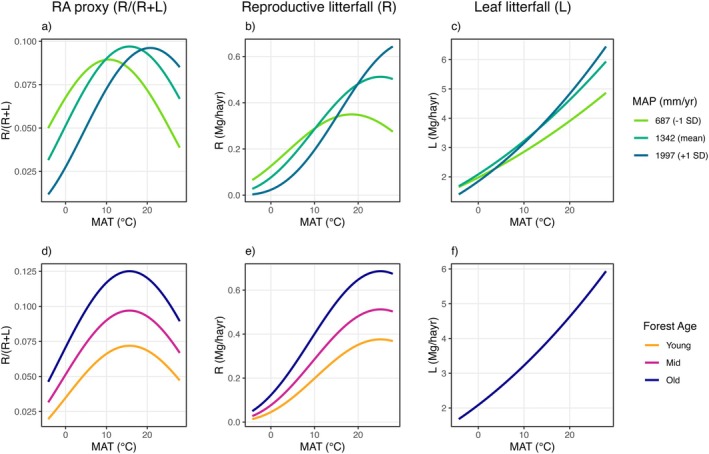
Predicted effects of climate terms (mean annual temperature (MAT), mean annual precipitation (MAP), and their quadratic terms and interaction; panels a‐c) and forest age (panels d‐f) on RA proxy (R/(R + L)), reproductive litterfall (R), and leaf litterfall (L) over mean annual temperature (MAT). Forest age is not included in the final model of leaf flux due to statistical non‐significance (panel f).

Our analyses revealed consistent positive relationships between stand age and reproductive investment. RA showed a significant positive response to forest age, increasing from young to old forest stands (Figures 3, 5, Table 1). This pattern is visible in the raw data for boreal and tropical forests, although not apparent in temperate forests (Figure 2c, Tables S12 and S13). The age‐related increase in RA was driven by a strong positive relationship between reproductive litterfall and stand age, while leaf litterfall showed no significant age‐related trends and the forest age term was removed from the final leaf litterfall model (Tables 1 and S4).

### 3.4 Variance Explained and Predictor Strength

The fixed effects in our linear mixed effects models explained only a moderate proportion of variation in our response variables, with marginal *R* ^2^ (fixed effects alone) values of 0.135 for RA, 0.283 for reproductive litterfall, and 0.500 for leaf litterfall models (Table 1).

Forest age emerged as a particularly robust predictor of RA with effect sizes comparable to climate variables, narrower confidence intervals, and higher significance levels (Figure 5, Table 1). Climate variables, while significant predictors, showed greater uncertainty in their effects.

## 4 Discussion

Our study presents the first global analysis of ecosystem‐scale RA in forests, revealing how environmental factors shape reproductive carbon allocation across diverse biomes. Using a novel proxy, R/(R + L), to quantify variation in RA at a global scale, we find that commonly used climate metrics (mean annual temperature, mean annual precipitation) are important but not the sole predictors of RA. Indicators of soil fertility also significantly affect RA, while forest age effects were comparable, and arguably more robust, than climate effects and showed an increasing trend with stand age.

We find evidence that RA and reproductive productivity are highest in tropical forests, supporting previous work analysing seed production (Journé et al. 2022; Moles et al. 2009). Interestingly, reproductive litterfall fluxes showed an approximately fivefold increase over temperature and moisture gradients (Figure 5b) – a more modest finding than the 250‐fold increase in community seed production reported by Journé et al. (2022). This discrepancy could arise from methodological differences. Beyond different response variables and data‐transformation approaches, the hierarchical state‐space model used by Journé et al. (2022) predicts seed production using fitted dispersal kernels, which may overestimate production when there are insufficient traps beneath tree crowns, a more common issue in species‐rich tropical forests (S.J. Wright, personal communication).

Increased RA in the tropics could be partially due to longer growing seasons, which may allow prolonged or more frequent fruit production in a year relative to total NPP (Hacket‐Pain et al. 2025; Wright and Calderón 2006). Increased tropical RA could also result from an adaptive response to intense species interactions, driven by high faunal and floral diversity, exerting strong positive selection pressure for high fruit and seed production (Dawkins and Krebs 1979; Journé et al. 2022). Greater reproductive productivity may ensure that a larger proportion of seeds escape predation or are dispersed to suitable environments for seedling establishment (Janzen 1971; Kolb et al. 2007).

Observed patterns in RA across biomes (boreal < temperate < tropical) may reflect differences in dominant plant types. Our analysis finds differences in RA by plant type that qualitatively align with our expectations, although only boreal needleleaf RA was statistically different from other plant types (Figure 2b, Tables S10 and S11). High site‐to‐site variation within dominant leaf morphology categories and inherent variability in reproductive output—both spatially and temporally—likely obscure more subtle plant type effects at the ecosystem‐level. Future analyses at the species level may provide additional insights into the influence of plant type on reproductive allocation.

### 4.1 Climate Effects

Our analysis finds concave down relationships between reproductive allocation and both mean annual temperature and precipitation: R/(R + L) peaks at approximately 18°C and 1500 mm annual precipitation (Figure 4a). This pattern represents a nearly 100% increase in RA from its lowest to highest point, reflecting a 500% increase in reproductive litterfall fluxes, compared to a more modest 200% increase in leaf litterfall fluxes over the same range (Figure 5a).

Reproductive and leaf litterfall fluxes showed contrasting responses to temperature extremes: reproductive litterfall declined at high temperatures, while leaf litterfall increased linearly with temperature (Figure 3b,c, Table 1). The decline in reproductive litterfall fluxes at high temperatures aligns with known constraints, including temperature‐mediated flower abortion, pollinator limitations, and reduced success under high moisture and cloud cover in tropical conditions (Detto et al. 2018; Iovane and Aronne 2022; Nussbaumer et al. 2020). In contrast, the positive effect of MAT on leaf litterfall mirrors previously observed patterns in NPP and ANPP (Del Grosso et al. 2008; Taylor et al. 2017).

These temperature sensitivities suggest a potential shift in reproductive‐vegetative growth balance under climate change, with recent studies already documenting climate‐driven changes in reproductive phenology and output (Buechling et al. 2016; Caignard et al. 2017; Hacket‐Pain and Bogdziewicz 2021). Free Air CO2 Enrichment (FACE) experiments document increasing fecundity in response to increasing CO2 concentrations (LaDeau and Clark 2001), with differential impacts across species expected to alter forest composition (Norby and Zak 2011; Stiling et al. 2004). Such changes could have cascading effects on ecosystem structure and function, highlighting the need to understand climate‐reproduction mechanisms and the response of RA to persistent change. Tools presented in this study can aid future investigations: the RA proxy R/(R + L) enables quantification of RA from standard litterfall data, while our global dataset provides benchmarks for vegetation and ecosystem model predictions across climate gradients.

### 4.2 Soil Fertility

While our analysis finds statistically significant relationships between RA and soil characteristics, particularly soil pH and soil N, robustness checks using on‐site soil data did not support these findings (Figure 3a, Table S14). Robustness checks are limited by small sample size, and do not fundamentally alter our main conclusions, but do suggest that caution is warranted when interpreting soil effects derived from SoilGrids, which may not capture important variables (e.g., total P) or local variation differing from large‐scale estimates (Poggio et al. 2021; de Sousa et al. 2020).

Interestingly, the relationship between soil characteristics and RA appears driven primarily by leaf litterfall responses (Figure 3c), with the highest leaf litterfall fluxes occurring in acidic, clay‐rich, high N soils typical of tropical forests. These patterns likely reflect broader ecosystem properties rather than soil characteristics alone. Our ability to fully assess soil‐RA relationships is limited by the lack of comprehensive phosphorus data outside the tropics. While other nutrients (Ca, B, Zn) may influence RA (Pandey 2018), future research should prioritise examining N and P relationships with reproductive allocation across forest types and climate gradients.

### 4.3 Forest Age

We expected higher RA in young and old forests relative to mid‐successional forests due to successional dynamics in light competition and species composition (Bazzaz 1979; Wright et al. 2005), but instead found a consistent increase in RA with forest age, with effect sizes rivalling those of climate predictors (Figure 3a). This pattern is evident in raw data from boreal and tropical forests, although not apparent in temperate forests, likely because temperate forest sites span a relatively broader climatic range (Figure 2c, Tables S12 and S13). The strength of forest age effects (narrow confidence intervals, high statistical significance) suggests that large‐scale patterns in RA over time are driven more strongly by the increasing proportion of mature trees as forests age than by changes in composition and light environments during early succession.

These findings appear to be at odds with evidence of declining fecundity during senescence (Qiu et al. 2021). However, both may be supported: even if fecundity declines, allocation to reproduction as a fraction of positive NPP could continue to increase. Older and/or larger trees generally face increasing hydraulic constraints and maintenance costs, reducing positive NPP available for reproduction and other functions (Mäkelä and Valentine 2001; Tang et al. 2014; Wenk et al. 2018). There is also evidence that the physiological costs of reproduction contribute to senescence, which is certainly the case for ~100 monocarpic species, which die after one reproductive event (Thomas 2011). Even if RA declines alongside fecundity as some species age, increasing forest RA with stand age may be driven by the larger influences of species turnover during succession and the nearly universal delay of RA until reproductive maturity (Bogdziewicz et al. 2020; Journé et al. 2024).

Several methodological considerations should be noted. Litterfall trap methods are rarely employed in young forests, and when used may underestimate reproductive productivity due to poor capture of small but reproductively active trees. Additionally, while age‐based patterns in RA are clear in raw data from boreal and tropical sites (Figure 2c), our broad age classification scheme may not adequately capture early successional dynamics, particularly in temperate forests where successional trajectories and timelines vary widely across ecosystems. More precise stand age data and ecosystem‐specific definitions of successional stages could better resolve these patterns.

Our results have important implications for vegetation demographic and ecosystem models, which typically allocate a static fraction of NPP to reproduction (Hanbury‐Brown et al. 2022). The strong relationship between forest age and RA highlights the need to incorporate age‐ or size‐dependent RA in vegetation models, allowing RA to increase as simulated plants or cohorts mature. Beyond demonstrating climate and age‐related patterns in RA, this study provides a globally applicable framework for incorporating field measures of reproductive dynamics into ecosystem models. The accompanying dataset can be used to benchmark RA predictions across diverse forest types, and the RA proxy we propose (R/(R + L)) can be easily constructed from litterfall observations. More realistic representation of reproductive dynamics could improve predictions of both carbon allocation and forest demographics, and enhance our understanding of how forest reproductive capacity responds to changing environmental conditions.

### 4.4 Variance Explained and Predictor Strength

The marginal *R* ^2^ values (*R* ^2^ = 0.135–0.5) in our models suggest substantial unexplained variation by our fixed effects. Additional variation may be explained by environmental factors not considered in this analysis (e.g., solar radiation, vapour pressure deficit), the limited temporal resolution of observations relative to reproductive variability and associated sampling error, and/or potential scale mismatches between site‐level measurements and broader‐scale climate and soil variables.

While we hypothesised that climate would primarily drive patterns in RA, our analysis revealed forest age as an equally important predictor. Climate relationships were complex, including both non‐linear responses and interactions between temperature and precipitation (Figures 3a and 5a,c). Despite this complexity, and though we were unable to test for age‐climate interactions, the magnitude of forest age coefficients, narrow confidence intervals, and strong statistical significance suggest that forest age rivals climate as a driver of ecosystem‐level reproductive allocation patterns (Table 1).

### 4.5 Limitations and Future Directions

Several methodological considerations warrant discussion. Reproductive litterfall is highly variable in space and time, ideally requiring long‐term observations. A lack of long‐term monitoring data likely drives biases in ecosystems dominated by masting species, where mast events can induce orders of magnitude differences in reproductive output among years (Hilton 2003; Müller‐Haubold et al. 2015). Particularly for systems with infrequent masting events, estimates of RA will be sensitive to the timing of measurements (Bogdziewicz et al. 2023; Fernández‐Martínez and Peñuelas 2021), and may also be skewed by publishing biases. We observed that studies carried out during non‐mast years were more likely to report only foliar litterfall, noting the minimal collection of reproductive structures in litterfall traps. These studies could not be included in our dataset, and the absence of these near‐zero measurements likely creates a positive bias, with published reproductive litterfall measurements over‐representing mast years. Temporal biases are likely compounded by spatial biases, as masting behaviour shows latitudinal patterns (Pearse et al. 2020).

While our approach represents a best available approximation, potential biases exist in the material collected by litterfall traps. Some species and reproductive structures are not well captured by litterfall traps, for example palms with large fronds. Furthermore, fruit and seed predators likely consume or remove much reproductive material before it can reach litterfall traps (Clark et al. 2001). Analysis of reproductive material in litterfall traps from Barro Colorado Island suggests that 31.8% of reproductive material is eaten or removed from litterfall, compared to approximately 9.7% of leaf material (Hanbury‐Brown et al. 2022). Additionally, our analysis does not account for resprouting and clonal reproduction, and thus underestimates total reproductive allocation, especially in systems where resprouting or clonal reproduction dominate, as is common in frequently disturbed environments (Barros et al. 2021; Bellingham and Sparrow 2000).

These limitations highlight the need for standardised, long‐term monitoring protocols that account for biases in litterfall trap data, quantify losses to consumers, and incorporate vegetative reproduction measurements. Such improvements would enhance our understanding of how RA patterns influence forest food webs and ecosystem dynamics under global change.

## 5 Conclusion

Our analysis provides the first global quantification of RA patterns across forests, revealing the primary role of climate and forest age as drivers of variation, and challenging the common assumption of globally fixed RA in vegetation models (Hanbury‐Brown et al. 2022; König et al. 2022). These patterns provide crucial empirical benchmarks for predictions of vegetation and plant growth, which have historically lacked data on RA across plant functional types and biomes. The observed variation in reproductive allocation has cascading implications for ecosystem function through modified trophic interactions, altered resource availability for consumers and seed dispersers, and shifts in forest regeneration patterns. As global change continues to alter environmental conditions worldwide, understanding these reproductive allocation patterns becomes essential for predicting forest responses and maintaining ecosystem services.

## Author Contributions

R.E.W. and L.M.K. conceptualised the study, R.E.W. synthesised data, performed analyses, and drafted the manuscript. L.M.K., H.C.M.‐L., H.Z.‐Z., J.B., E.B. and M.R.S. contributed to revisions. H.Z.‐Z., K.A., S.A.‐B., L.A., A.B., J.B., E.B., L.C.‐R., P.C., C.A.L.D., E.C.N., B.d.O.S., W.F.‐R., J.N.F., R.F., C.G., W.H.H., C.A.J., Y.M., B.M., B.H.M.J., A.C.M., K.d.S.P., S.R., T.R., N.S., M.S. and M.R.S. provided data.

## Peer Review

The peer review history for this article is available at https://www.webofscience.com/api/gateway/wos/peer‐review/10.1111/ele.70191.

## Supporting information


**Data S1:** ele70191‐sup‐0001‐Supinfo.pdf.

## Data Availability

The data and code supporting the findings of this study are openly available in the NGEE‐Tropics portal on the ESS‐DIVE data repository at https://doi.org/10.15485/2523672.
