# Supplementary material for: Forest Age Rivals Climate to Explain Reproductive Allocation Patterns in Forest Ecosystems Globally
Source: Ecol Lett. 2025 Aug 25;28(8):e70191. doi: 10.1111/ele.70191 (PMC12377453; doi:10.1111/ele.70191)
Supplement: Supplementary file 1 — Data S1: ele70191‐sup‐0001‐Supinfo.pdf. [file ELE-28-0-s001.pdf]

Supporting information

Title: Forest age rivals climate to explain reproductive allocation patterns in forest ecosystems globally

Authors:

Ward, Rachel E.[1], Zhang-Zheng, Huanyuan[2], Abernethy, Kate[3,4], Adu-Bredu, Stephen[5], Arroyo, Luzmilla[6], Bailey, Andrew[7], Barlow, Jos[8], Berenguer, Erika[2,8], Chesini-Rossi, Liana[9], Cho, Percival[10], Dahlsjö, Cecilia A. L.[2], das Neves, Eder Carvalho[11], de Oliveira Sales, Bianca[12], Farfan-Rios, William[13,14], Ferreira, Joice Nunes[15], Freitag, Renata[16], Girardin, Cécile[17], Huaraca Huasco, Walter[2], Joly, Carlos A.[18], Malhi, Yadvinder[2], Marimon, Beatriz[11], Marimon Junior, Ben Hur[11], Morel, Alexandra C.[19], Muller-Landau, Helene C.[20], Peixoto, Karine da Silva[12], Reis, Simone[11,21], Riutta, Terhi [2], Salinas, Norma[22], Seixas, Marina[14], Silman, Miles R.[12,13], Kueppers, Lara M.[1,23]

Methods S1:

To assess the robustness of our findings related to soil characteristics, we conducted additional analyses using subsets of sites with on-site soil measurements, as opposed to more inherently uncertain estimates from SoilGrids250, which were used in our main analysis.

We performed the following analyses:

1. Using data from 67 sites with on-site measurements of soil texture, pH, cation exchange capacity (CEC), and nitrogen (N), we refit our full model (see Methods S2) for our reproductive allocation (RA) proxy R/(R+L), reproductive litterfall (R), and leaf litterfall (L) response variables. This analysis tested the robustness of our primary findings.
2. While total phosphorus (P) was unavailable for all sites in our main dataset, we obtained on-site measurements for 34 sites, primarily located in tropical regions where total P is hypothesized to limit NPP. Of these, 20 sites also had complete data for on-site measurements of soil texture, pH, CEC, and N. Using this limited subset, we fit an exploratory model including total P, acknowledging its low statistical power due to the small sample size.
3. To further investigate the potential role of phosphorus, we examined correlations between total P and R/(R+L), R, and L across all 34 sites where total P was reported.

These robustness checks and exploratory analyses provide additional context for interpreting our main results and highlight the need for more comprehensive soil data in future studies.

Methods S2:

The full structure of our linear mixed-effects statistical model was:

$$\left(\frac{R}{R+L}\right)_{ij} = \beta_0 + \beta_1 MAT + \beta_2 MAT^2 + \beta_3 MAP + \beta_4 MAP^2 + \beta_5 MAT * MAP + \beta_6 soil\,pH + \beta_7 soil\,N + \beta_8 soil\,CEC + \beta_9\, soil\,texture + \beta_{10}\, MAP * soil\,texture + \beta_{11}\, forest\,age + \mu_i + \varepsilon_{ij} \quad \text{(Eq. S1)}$$

Where:

$\mu_i \sim N(0, \sigma^2{}_{\mathrm{u}})$ represents the random site effect

$\varepsilon_{ij} \sim N(0, \sigma^2/w_{ij})$ represents the residual error

$w_{ij} = \sqrt{Duration_{ij}}$ is the variance weight for observation *j* at site *i*

We fit parallel models for the Box-Cox transformed response variables RA proxy (R/(R+L)), reproductive litterfall (R) and leaf litterfall (L); see Table S1 and S2 for descriptions and summary statistics of response variables and independent variables, respectively.

We plotted model residuals against predictor variables to assess whether the assumed functional relationships of MAT and MAP were appropriate for all response variables (Figures S9-S11). The MAT^2 term was not supported in the leaf litterfall flux model and so was removed. The absence of systematic patterns in residual plots support our choice of functional form in the final models, with no evidence of unexplained nonlinear relationships.

Models include “site” as a random intercept, which we defined as a unique set of geographic coordinates. Observations were weighted by the square root of sampling

duration (years) to reflect the increased reliability of longer-term studies while not overemphasizing a few individual sites. For sites from data papers that did not report sampling duration (n = 56), we assumed one year duration based on the minimum inclusion criterion of data papers.

To assess potential multicollinearity, we calculated the variance inflation factor for all covariates (VIF). While MAT and MAP are correlated across global climate gradients (r=0.74, Figure S5), we retained both variables as they represent distinct mechanisms that may influence RA. Despite a modest correlation between MAP and soil pH (r = -0.64, Figure S1), VIF were acceptable (<5) for all main effect terms. As expected, higher VIF values were observed for the MAT:MAP interaction term and squared covariate terms in all models (Figures S6-S8).

We assessed model assumptions of normally distributed residuals via Q-Q and residual vs. fitted value plots. While Q-Q plots show deviations from the 1:1 line at the extremes, linear mixed-effects models have been shown to be robust to violations of distributional assumptions (Schielzeth et al. 2020). Visual assessment of residual vs. fitted values plots shows no systematic changes in residual spread, supporting the assumption of homoscedasticity (Figure S6-S8).

Table S1. Description of response variables and summary statistics for each (n = 824)

Variable	Description	Units	Range	Mean	Standard deviation
R	Reproductive material litterfall flux (fruits, flowers, seeds, cones, bracts)	Mg/ha year	(0.002 - 3.51)	0.584	0.554
L	Leaf and other foliar material litterfall flux	Mg/ha year	(0.044 – 20.7)	4.11	2.22
R/(R+L)	Proxy for reproductive allocation (R/NPP)	-	(0.001 – 0.546)	0.119	0.082

Table S2. Description of numeric predictor variables and summary statistics for each (n = 824)

Variable	Description	Units	Range	Mean	Standard deviation	Source	Reference
MAT	mean annual temperature (1970-2000)	degrees C	(-4.27 – 28.0)	14.7	8.24	WorldClim2	Fick and Hijmans (2017)
MAP	mean annual precipitation (1970-2000)	mm/year	(199 - 3554)	1342.02	654.72	WorldClim2	Fick and Hijmans (2017)
Soil N	total soil nitrogen	g/kg	(0.57 - 4)	1.72	0.63	SoilGrids2.0	Poggio et al. (2021)
Soil pH	soil pH	pH	(3.95 - 8.13)	5.47	0.75	SoilGrids2.0	Poggio et al. (2021)
Soil CEC	soil cation exchange capacity	cmol(c)/kg	(4 - 44.65)	14.98	5.83	SoilGrids2.0	Poggio et al. (2021)
Sand	soil percent sand	%	(4.15 - 95.22)	39.15	16.39	SoilGrids2.0	Poggio et al. (2021)
Clay	percent clay	%	(1.55 – 78.78)	23.44	10.36	SoilGrids2.0	Poggio et al. (2021)
Silt	percent silt	%	(2.4 - 73.28)	28.35	12.84	SoilGrids2.0	Poggio et al. (2021)
Soil texture index	log (% sand / % clay)	-	(-2.43 - 4.12)	0.52	0.85	SoilGrids2.0	Poggio et al. (2021)

Table S3. Forest successional stage and age classification in dataset (n = 290 sites where both successional stage and age are reported)

Forest biome	Forest stage	Reported age range (years)	Observations
Boreal	late-successional	65 – 210	4
Boreal	old-growth	114 – 400	5
Boreal	secondary	30 – 95	12
Temperate	late-successional	60 - 90	6
Temperate	old-growth	100 - 1000	46
Temperate	secondary	15 – 200	132
Tropical	early-successional	8 - 10	3
Tropical	mid-successional	35 - 50	2
Tropical	old-growth	100 - 400	23
Tropical	secondary	5 - 140	57

Table S4. Full model coefficients, confidence intervals (CI) and p-values for response variables RA proxy (R/(R+L)), reproductive litterfall (R), and leaf litterfall (L). Prior to model fitting, all response variables were Box-Cox transformed, and all numerical variables were scaled and centered (MAT = mean annual temperature, MAP = mean annual precipitation).

	**RA proxy (R/(R+L))**			**Reproductive litterfall (R)**			**Leaf litterfall (L)**		
Predictors	*Estimates*	*CI*	*p*	*Estimates*	*CI*	*p*	*Estimates*	*CI*	*p*
(Intercept)	-1.76	-1.83 – -1.68	**<0.001**	-1.35	-1.53 – -1.17	**<0.001**	1.85	1.65 – 2.04	**<0.001**
MAT	0.24	0.03 – 0.44	**0.025**	0.58	0.08 – 1.07	**0.022**	0.11	-0.44 – 0.66	0.693
MAT2	-0.21	-0.38 – -0.04	**0.016**	-0.33	-0.74 – 0.08	0.113	0.38	-0.07 – 0.83	0.100
MAP	0.16	-0.03 – 0.36	0.106	0.71	0.23 – 1.18	**0.003**	0.46	-0.07 – 0.99	0.086
MAP2	-0.19	-0.38 – -0.01	**0.040**	-0.73	-1.18 – -0.29	**0.001**	-0.36	-0.86 – 0.13	0.148
Soil pH	0.08	0.03 – 0.12	**0.001**	0.07	-0.03 – 0.17	0.169	-0.18	-0.29 – -0.07	**0.002**
Soil N	-0.06	-0.10 – -0.02	**0.004**	-0.08	-0.17 – 0.02	0.127	0.13	0.02 – 0.24	**0.019**
Soil texture index	0.00	-0.03 – 0.03	0.999	-0.08	-0.16 – -0.01	**0.031**	-0.17	-0.25 – -0.09	**<0.001**
Soil CEC	-0.02	-0.05 – 0.02	0.291	-0.06	-0.15 – 0.02	0.142	-0.08	-0.18 – 0.01	0.079
Forest age [mid]	0.11	0.05 – 0.17	**<0.001**	0.27	0.13 – 0.41	**<0.001**	-0.01	-0.15 – 0.13	0.861
Forest age [old]	0.22	0.16 – 0.29	**<0.001**	0.54	0.38 – 0.69	**<0.001**	0.08	-0.08 – 0.24	0.334
MAT × MAP	0.07	0.00 – 0.15	**0.049**	0.18	0.00 – 0.35	**0.046**	-0.04	-0.23 – 0.15	0.697
MAP × Soil texture index	-0.04	-0.07 – -0.01	**0.014**	-0.06	-0.13 – 0.02	0.141	0.07	-0.01 – 0.16	0.102
**Random Effects**									
σ^2	0.04			0.20			0.18		
τ_{00}	0.04 $_{site}$			0.26 $_{site}$			0.38 $_{site}$		
ICC	0.53			0.57			0.68		
N	393 $_{site}$			393 $_{site}$			393 $_{site}$		
Observations	824			824			824		
Marginal R^2 / Conditional R^2	0.138 / 0.593			0.297 / 0.698			0.512 / 0.844		
AIC	184.472			1575.100			1614.237		

Table S5. Model comparison of the final model (Table 1) fit for response variable RA proxy (R/(R+L)) using alternate weighting schemes in the linear mixed effects model described in Methods S2 (sqrt(Duration) = square root of sampling duration (years); Duration = sampling duration (years); no weight = data points have equal weight).

	**sqrt(Sampling duration)**			**Sampling duration**			**no weight**		
Predictors	*Estimates*	*CI*	*p*	*Estimates*	*CI*	*p*	*Estimates*	*CI*	*p*
(Intercept)	-1.76	-1.84 – -1.69	**<0.001**	-1.78	-1.86 – -1.70	**<0.001**	-1.75	-1.82 – -1.68	**<0.001**
MAT	0.24	0.04 – 0.45	**0.021**	0.26	0.04 – 0.49	**0.021**	0.23	0.03 – 0.42	**0.022**
MAT2	-0.21	-0.38 – -0.04	**0.015**	-0.23	-0.41 – -0.05	**0.015**	-0.20	-0.36 – -0.04	**0.015**
MAP	0.18	-0.02 – 0.37	0.072	0.19	-0.02 – 0.39	0.071	0.17	-0.02 – 0.35	0.085
MAP2	-0.21	-0.39 – -0.03	**0.025**	-0.22	-0.42 – -0.03	**0.023**	-0.19	-0.37 – -0.02	**0.032**
Soil pH	0.07	0.03 – 0.12	**0.001**	0.08	0.03 – 0.12	**0.001**	0.07	0.03 – 0.11	**0.001**
Soil N	-0.07	-0.10 – -0.03	**0.001**	-0.06	-0.11 – -0.02	**0.003**	-0.07	-0.10 – -0.03	**<0.001**
Soil texture index	0.00	-0.03 – 0.03	0.911	0.01	-0.03 – 0.04	0.654	-0.00	-0.03 – 0.02	0.762
Forest age [mid]	0.12	0.06 – 0.18	**<0.001**	0.12	0.06 – 0.18	**<0.001**	0.11	0.05 – 0.17	**<0.001**
Forest age [old]	0.22	0.16 – 0.29	**<0.001**	0.24	0.17 – 0.30	**<0.001**	0.21	0.15 – 0.28	**<0.001**
MAT × MAP	0.08	0.01 – 0.15	**0.032**	0.09	0.01 – 0.17	**0.030**	0.07	0.00 – 0.14	**0.037**
MAP × Soil texture index	-0.04	-0.07 – -0.01	**0.011**	-0.04	-0.08 – -0.01	**0.014**	-0.04	-0.07 – -0.01	**0.012**
**Random Effects**									
σ^2	0.04			0.03			0.04		
τ_{00}	0.04 $_{site}$			0.05 $_{site}$			0.04 $_{site}$		
ICC	0.53			0.57			0.50		
N	393 $_{site}$			393 $_{site}$			393 $_{site}$		
Observations	824			824			824		
Marginal R^2 / Conditional R^2	0.135 / 0.592			0.146 / 0.629			0.120 / 0.560		
AIC	183.613			258.219			140.821		

Table S6. Candidate forest age classification schemes (as defined by “mid” successional age range). Scheme 4 was used in the analysis presented in the main text.

Scheme	Rationale	Biome	“Mid” age range
1	Control	tropical	50-100
		temperate	50-100
		boreal	50-100
2	Tropical forest succession is fast relative to temperate and boreal forests	tropical	20-60
		temperate	50-100
		boreal	50-100
3	Successional stages differ by age for tropical, temperate, and boreal forests	tropical	20-60
		temperate	40-80
		boreal	50-100
4	Successional stages differ by age for tropical, temperate, and boreal forests	tropical	20-60
		temperate	40-100
		boreal	50-120
5	Successional stages differ by age for tropical, temperate, and boreal forests	tropical	30-60
		temperate	40-80
		boreal	50-100

Table S7. Comparison of the final model (Table 1) fit for the RA proxy (R/(R+L)) using alternate forest age classification schemes, described in Table S6. Scheme 4 was used in the analysis presented in the main text.

	Scheme 1			Scheme 2			Scheme 3			Scheme 4			Scheme 5		
Predictors	*Estimates*	*CI*	*p*	*Estimates*	*CI*	*p*	*Estimates*	*CI*	*p*	*Estimates*	*CI*	*p*	*Estimates*	*CI*	*p*
(Intercept)	-1.72	-1.79 – -1.65	**<0.001**	-1.73	-1.80 – -1.65	**<0.001**	-1.76	-1.83 – -1.68	**<0.001**	-1.76	-1.83 – -1.68	**<0.001**	-1.75	-1.82 – -1.67	**<0.001**
MAT	0.25	0.04 – 0.45	**0.018**	0.25	0.04 – 0.45	**0.018**	0.23	0.03 – 0.44	**0.024**	0.24	0.03 – 0.44	**0.024**	0.23	0.03 – 0.43	**0.026**
MAT2	-0.21	-0.38 – -0.04	**0.013**	-0.22	-0.39 – -0.05	**0.012**	-0.20	-0.37 – -0.03	**0.018**	-0.21	-0.38 – -0.04	**0.016**	-0.20	-0.37 – -0.03	**0.022**
MAP	0.16	-0.03 – 0.35	0.096	0.16	-0.03 – 0.35	0.090	0.16	-0.03 – 0.35	0.100	0.16	-0.03 – 0.35	0.094	0.15	-0.04 – 0.34	0.113
MAP2	-0.20	-0.38 – -0.02	**0.033**	-0.20	-0.38 – -0.02	**0.030**	-0.19	-0.37 – -0.01	**0.036**	-0.19	-0.38 – -0.01	**0.034**	-0.19	-0.37 – -0.01	**0.041**
Soil pH	0.07	0.03 – 0.11	**<0.001**	0.07	0.03 – 0.11	**<0.001**	0.07	0.03 – 0.11	**<0.001**	0.08	0.04 – 0.12	**<0.001**	0.07	0.03 – 0.11	**<0.001**
Soil N	-0.05	-0.09 – -0.01	**0.008**	-0.06	-0.10 – -0.02	**0.005**	-0.06	-0.10 – -0.02	**0.005**	-0.06	-0.10 – -0.02	**0.003**	-0.06	-0.10 – -0.02	**0.005**
Soil CEC	-0.02	-0.05 – 0.02	0.266	-0.02	-0.05 – 0.02	0.281	-0.02	-0.05 – 0.02	0.266	-0.02	-0.05 – 0.02	0.288	-0.02	-0.05 – 0.02	0.267
Forest age [mid]	0.08	0.03 – 0.14	**0.004**	0.07	0.02 – 0.13	**0.013**	0.11	0.05 – 0.17	**0.001**	0.11	0.05 – 0.17	**<0.001**	0.10	0.04 – 0.16	**0.001**
Forest age [old]	0.17	0.11 – 0.23	**<0.001**	0.19	0.13 – 0.25	**<0.001**	0.21	0.15 – 0.28	**<0.001**	0.22	0.16 – 0.29	**<0.001**	0.20	0.14 – 0.27	**<0.001**
MAT × MAP	0.08	0.00 – 0.15	**0.037**	0.08	0.01 – 0.15	**0.033**	0.08	0.00 – 0.15	**0.039**	0.07	0.00 – 0.14	**0.046**	0.07	0.00 – 0.14	**0.044**
MAP × Soil texture index	-0.04	-0.07 – -0.00	**0.026**	-0.04	-0.07 – -0.01	**0.019**	-0.04	-0.07 – -0.01	**0.020**	-0.04	-0.07 – -0.01	**0.013**	-0.04	-0.07 – -0.01	**0.022**
**Random Effects**															
σ^2	0.04			0.04			0.04			0.04			0.04		
τ_{00}	0.04 $_{site}$			0.04 $_{site}$			0.04 $_{site}$			0.04 $_{site}$			0.04 $_{site}$		
ICC	0.51			0.52			0.52			0.53			0.52		
N	393 $_{site}$			393 $_{site}$			393 $_{site}$			393 $_{site}$			393 $_{site}$		
Observations	824			824			824			824			824		
Marginal R^2 / Conditional R^2	0.126 / 0.576			0.133 / 0.586			0.139 / 0.588			0.139 / 0.593			0.137 / 0.585		
AIC	192.895			188.287			184.517			182.460			186.276		

Table S8. Analysis of variance (ANOVA) results comparing the RA proxy R/(R+L) across forest biomes using Box-Cox transformed data.

Source	Df	Sum.Sq	Mean.Sq	F.value	P.value
Biome	2	1.991	0.995	9.846	5.95e-05
Residuals	821	82.997	0.101		NA

Table S9. Post-hoc Tukey's HSD test results showing pairwise comparisons of the RA proxy R/(R+L) between forest biomes using Box-Cox transformed data.

Comparison	Difference	Lower CI	Upper CI	P value
Temperate-Boreal	0.139	0.021	0.257	0.016
Tropical-Boreal	0.205	0.085	0.325	<2e-16
Tropical-Temperate	0.066	0.012	0.120	0.012

Table S10. Analysis of variance (ANOVA) results comparing the RA proxy R/(R+L) across plant functional types using Box-Cox transformed data.

Source	Df	Sum.Sq	Mean.Sq	F.value	P.value
Plant type	3	2.089	0.696	6.653	0.000196
Residuals	712	74.523	0.105		NA

Table S11: Post-hoc Tukey's HSD test results showing pairwise comparisons between the RA proxy R/(R+L) across plant functional types using Box-Cox transformed data.

Comparison	Difference	Lower CI	Upper CI	P value
Temperate needleleaf-Boreal needleleaf	0.180	0.019	0.342	0.022
Temperate broadleaf-Boreal needleleaf	0.192	0.038	0.347	0.008
Tropical broadleaf-Boreal needleleaf	0.249	0.096	0.401	<2e-16
Temperate broadleaf-Temperate needleleaf	0.012	-0.078	0.101	0.986
Tropical broadleaf-Temperate needleleaf	0.068	-0.018	0.154	0.173
Tropical broadleaf-Temperate broadleaf	0.056	-0.015	0.128	0.181

Table S12. Analysis of variance (ANOVA) results comparing the RA proxy R/(R+L) between forest age groups within biome using Box-Cox transformed data.

Biome	Source	Df	Sum.Sq	Mean.Sq	F.value	P.value
Boreal	Forest_age_group	2	1.785	0.892	16.157	6.7e-06
Boreal	Residuals	41	2.265	0.055		NA
Temperate	Forest_age_group	2	0.252	0.126	1.132	0.323
Temperate	Residuals	445	49.592	0.111		NA
Tropical	Forest_age_group	2	1.300	0.650	7.691	0.000544
Tropical	Residuals	329	27.803	0.085		NA

Table S13. Post-hoc Tukey's HSD test results showing pairwise comparisons of the RA proxy R/(R+L) between forest age groups within biome where indicated by ANOVA ($p < 0.01$) using Box-Cox transformed data.

Biome	Comparison	Difference	Lower CI	Upper CI	P value
Boreal	mid-young	0.317	0.119	0.515	0.001
Boreal	old-young	0.584	0.327	0.840	<2e-16
Boreal	old-mid	0.267	0.032	0.501	0.023
Tropical	mid-young	0.139	-0.014	0.293	0.084
Tropical	old-young	0.220	0.075	0.364	0.001
Tropical	old-mid	0.080	-0.003	0.164	0.064

Table S14. Model summary (full model) for response variables RA proxy (R/(R+L)), reproductive litterfall (R) and leaf litterfall (L) fit with a subset of data (n=67) where on-site soil characteristics were available. “Mid”-aged forests are the reference level for forest age effects because of the absence of “young” sites in this dataset.

	**R/(R+L); field sampled soil**			**R; field sampled soil**			**L; field sampled soil**		
Predictors	*Estimates*	*CI*	*p*	*Estimates*	*CI*	*p*	*Estimates*	*CI*	*p*
(Intercept)	-1.46	-1.58 – -1.34	**<0.001**	-1.45	-2.01 – -0.89	**<0.001**	1.75	1.17 – 2.33	**<0.001**
MAT	0.59	0.20 – 0.97	**0.004**	2.55	0.72 – 4.38	**0.007**	2.12	0.21 – 4.03	**0.031**
MAT2	-0.45	-0.78 – -0.11	**0.010**	-1.91	-3.50 – -0.32	**0.020**	-1.47	-3.14 – 0.21	0.085
MAP	-0.35	-0.73 – 0.03	0.071	-0.18	-1.99 – 1.63	0.844	2.43	0.52 – 4.34	**0.014**
MAP2	0.24	-0.13 – 0.60	0.197	-0.06	-1.77 – 1.66	0.946	-2.16	-3.97 – -0.35	**0.021**
soil pH	0.01	-0.05 – 0.07	0.755	0.02	-0.28 – 0.33	0.878	-0.13	-0.46 – 0.20	0.436
soil N	-0.01	-0.08 – 0.07	0.840	0.07	-0.28 – 0.42	0.690	0.09	-0.27 – 0.46	0.612
soil texture index	0.03	-0.02 – 0.08	0.262	-0.02	-0.25 – 0.22	0.878	-0.33	-0.57 – -0.08	**0.010**
soil CEC	0.03	-0.05 – 0.11	0.397	-0.02	-0.39 – 0.36	0.934	-0.20	-0.59 – 0.19	0.310
Forest age [old]	0.08	-0.01 – 0.18	0.086	0.29	-0.17 – 0.75	0.193	-0.04	-0.37 – 0.29	0.798
MAT × MAP	0.06	-0.08 – 0.20	0.371	0.40	-0.27 – 1.06	0.234	0.67	-0.03 – 1.36	0.059
MAP × soil texture index	-0.02	-0.05 – 0.02	0.441	-0.03	-0.21 – 0.16	0.773	0.05	-0.14 – 0.25	0.588
**Random Effects**									
σ^2	0.00			0.09			0.02		
τ_{00}	0.02 site			0.41 site			0.62 site		
ICC	0.77			0.82			0.98		
N	56 site			56 site			56 site		
Observations	67			67			67		
Marginal R^2 / Conditional R^2	0.350 / 0.850			0.343 / 0.885			0.626 / 0.991		
AIC	-47.730			158.301			145.753		

Figure S1. Correlation between log(R/(R+L)) and log(R/NPP) (R = reproductive, L = leaf litterfall, NPP = net primary productivity) for sites reported in Figure 1a.

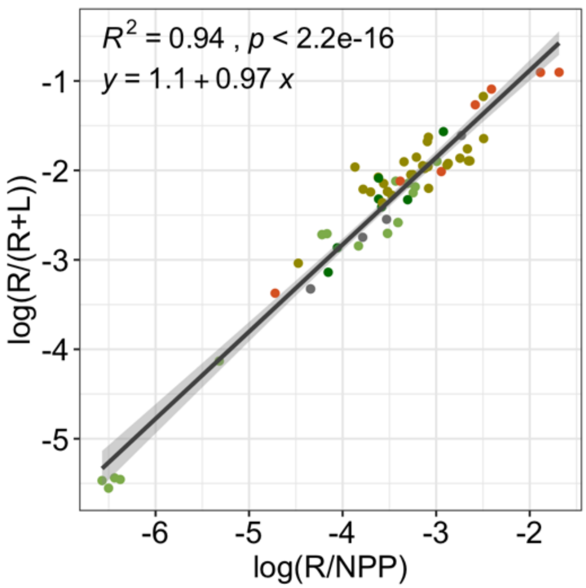

Figure S2. Correlation between log(R+L) and log(NPP) (R = reproductive, L = leaf litterfall, NPP = net primary productivity) for sites reported in Figure 1a.

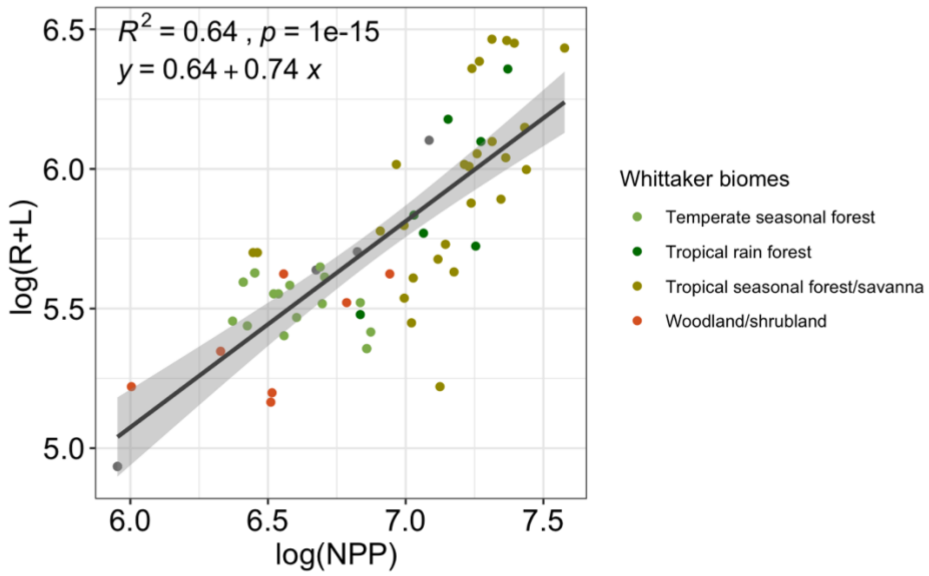

Figure S3. Distribution of sampling duration (years) across all study sites (n=824)

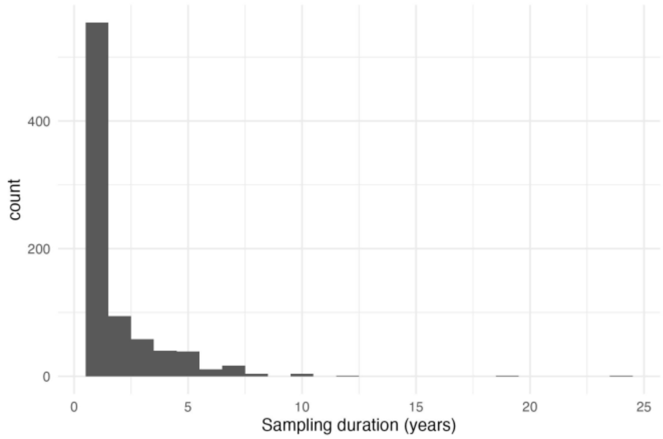

Figure S4. Histograms showing raw and Box-Cox transformed response variable

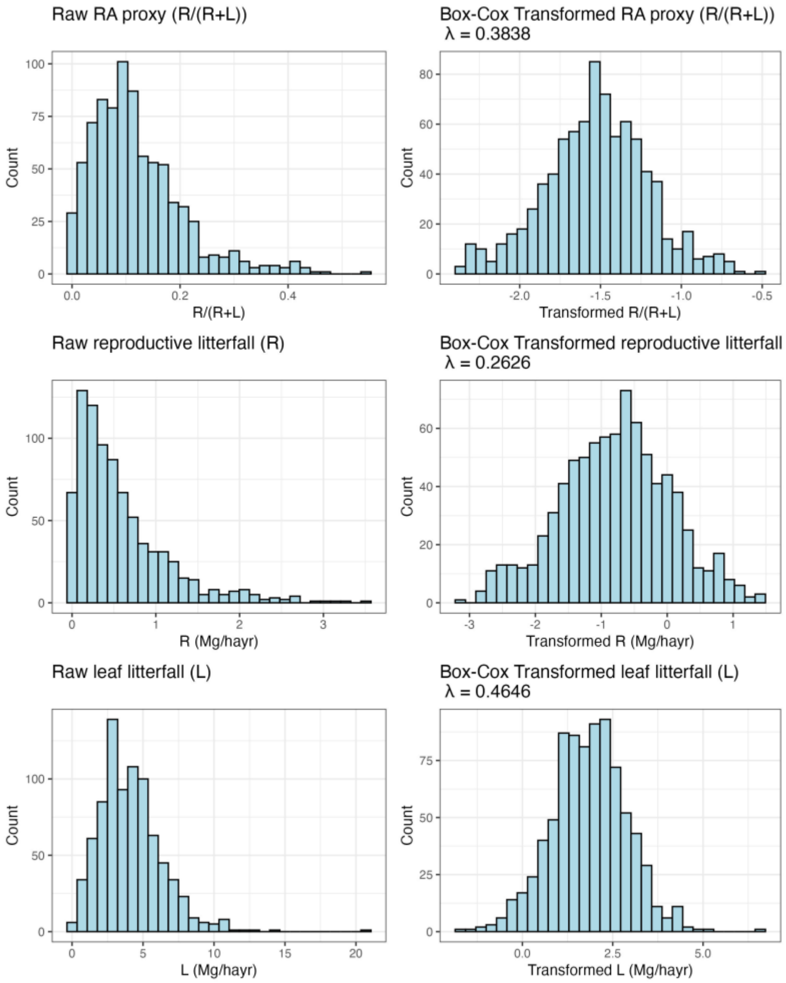

Figure S5. Correlation (r) matrix for potential covariates (statistically significant correlations shown)

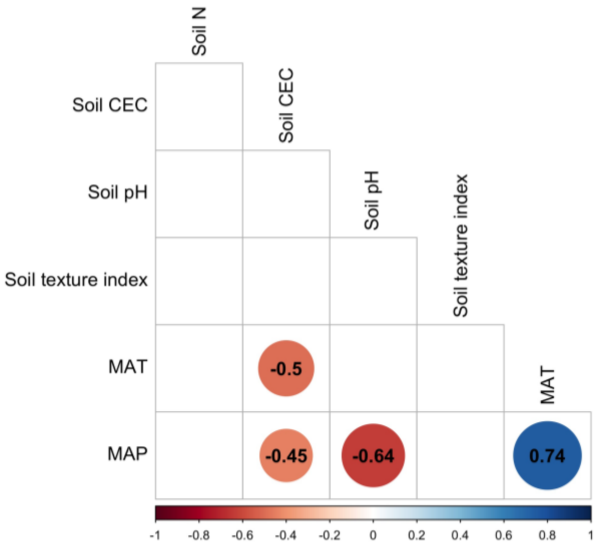

Figure S6. Diagnostic plots for final model of RA proxy (R/(R+L))

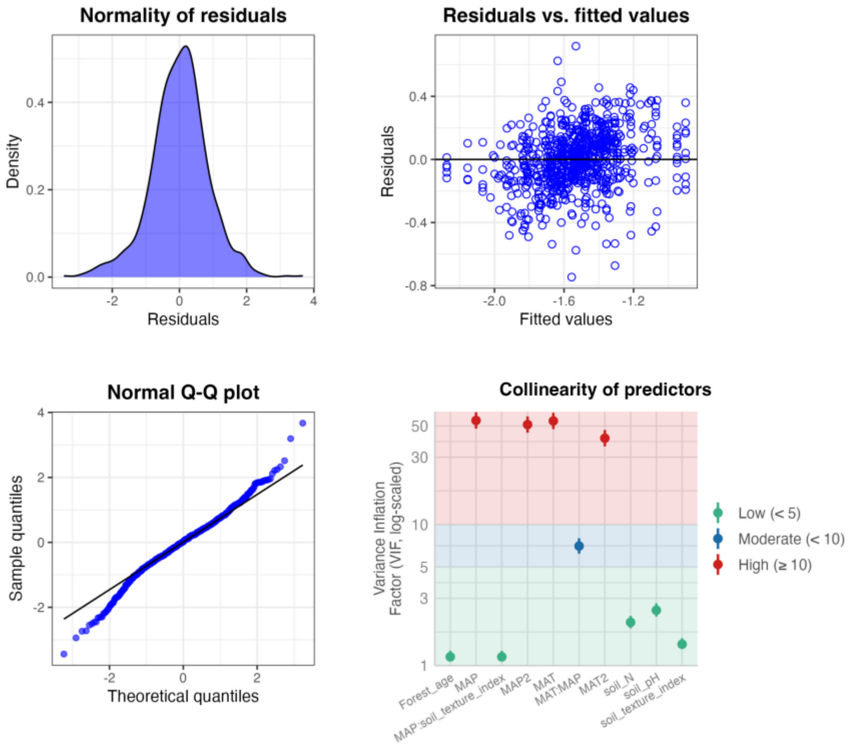

Figure S7. Diagnostic plots for final model of reproductive litterfall (R)

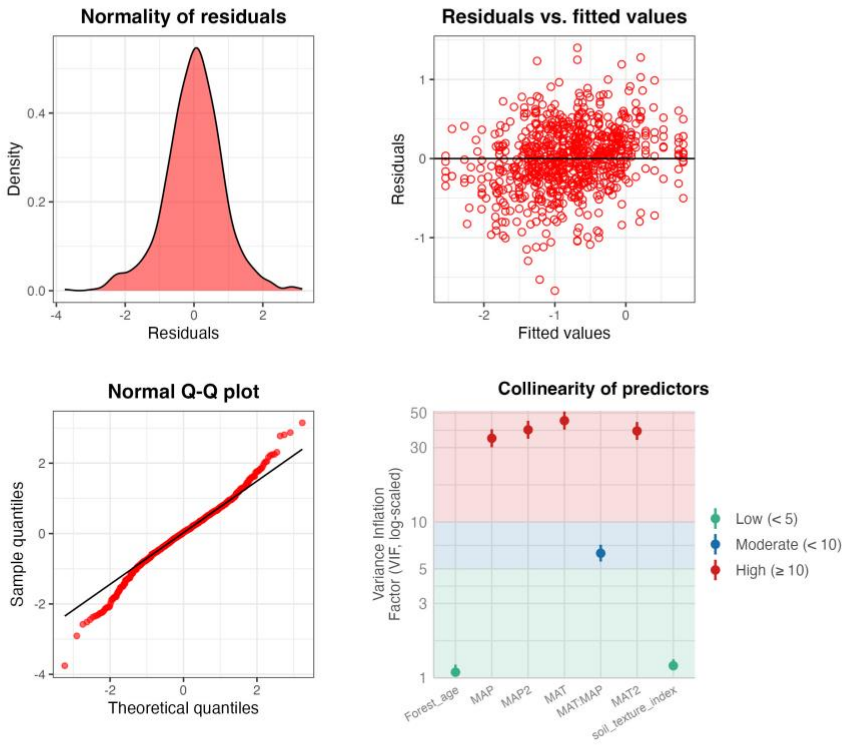

Figure S8. Diagnostic plots for final model of leaf litterfall (L)

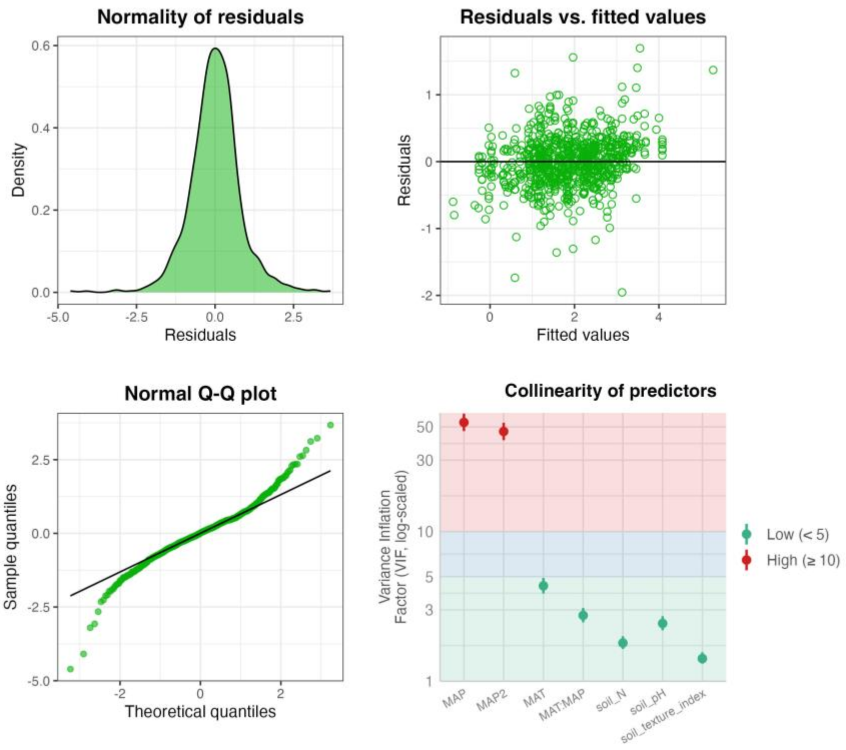

Figure S9. Model residuals vs. independent variables for final model of RA proxy (R/(R+L))

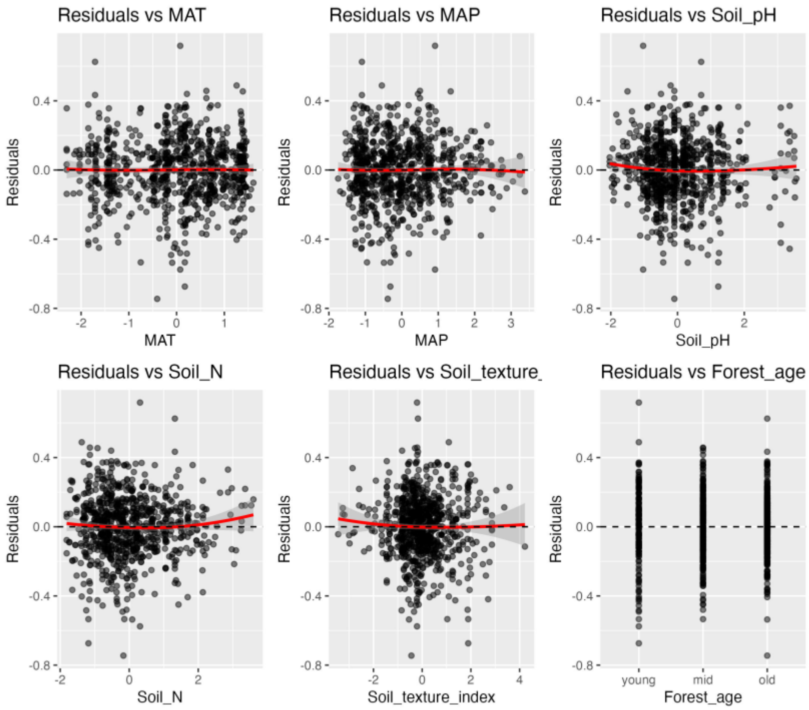

Figure S10. Model residuals vs. independent variables for final model of reproductive litterfall (R)

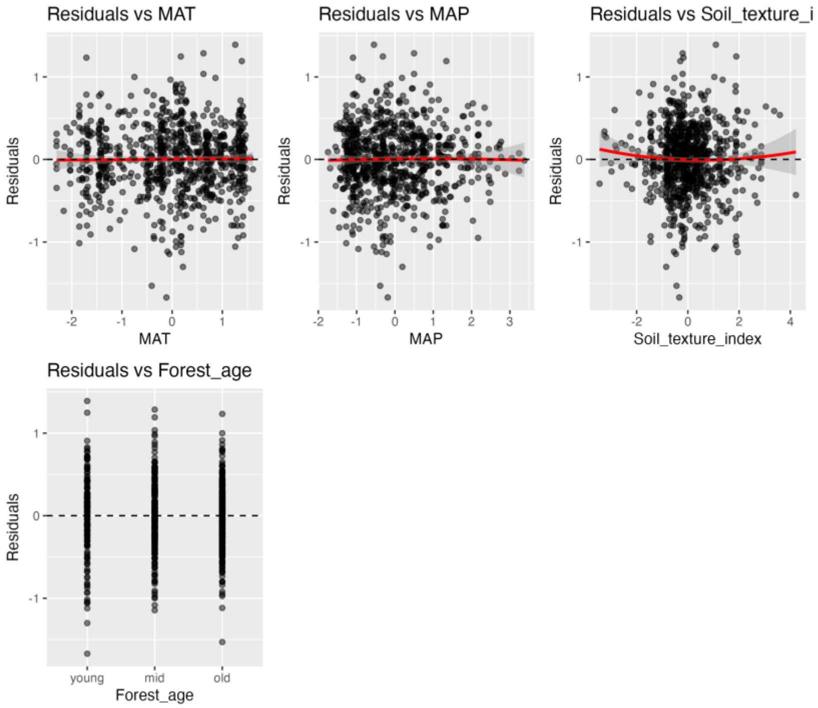

Figure S11. Model residuals vs. independent variables for final model of leaf litterfall (L)

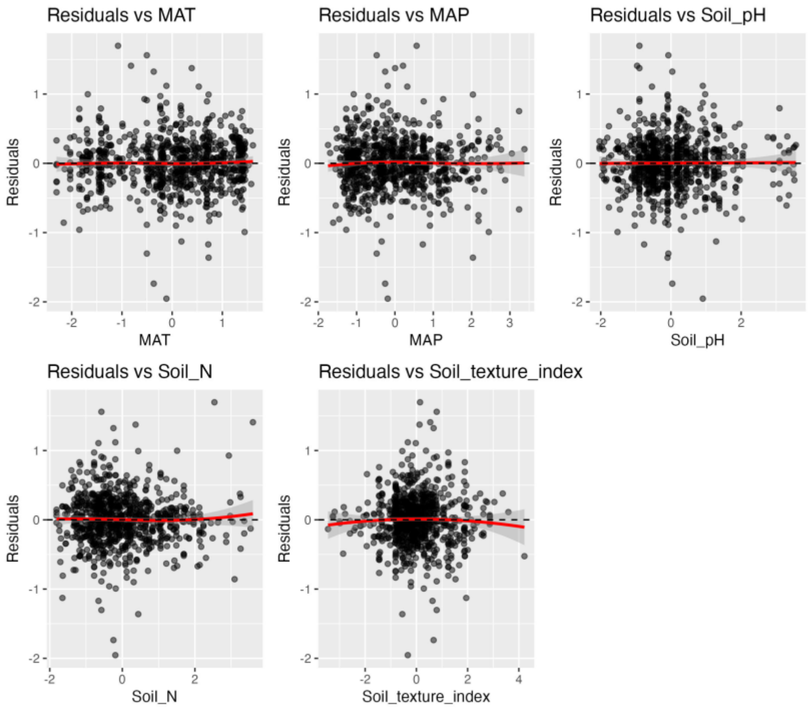

Figure S12. Correlation between RA proxy (R/(R+L)), reproductive litterfall (R) and leaf litterfall (L) and total phosphorus (n = 34)

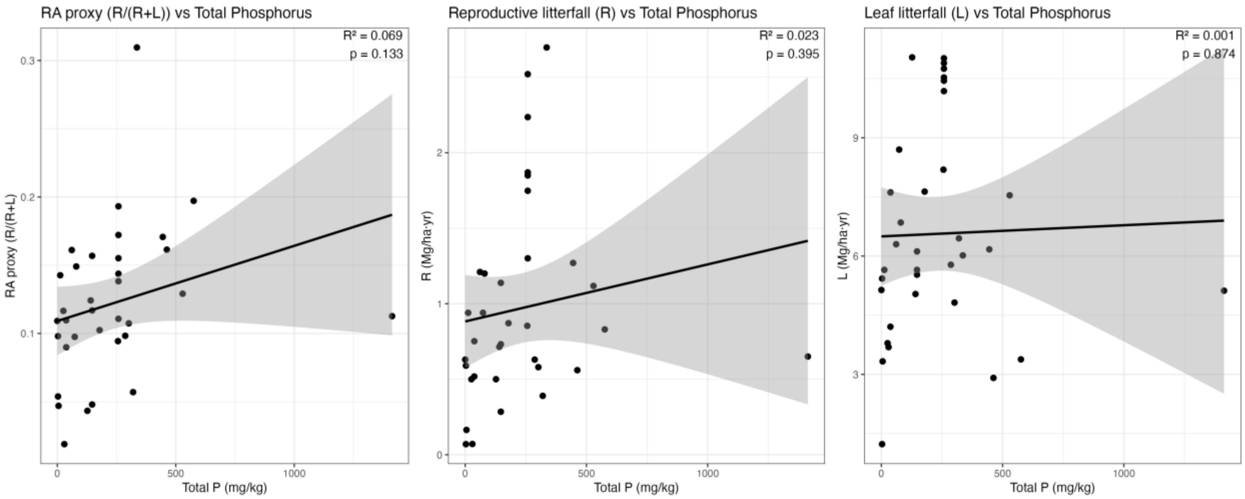

References

Fick, Stephen E., and Robert J. Hijmans. 2017. “WorldClim 2: New 1-km Spatial Resolution Climate Surfaces for Global Land Areas.” *International Journal of Climatology: A Journal of the Royal Meteorological Society* 37 (12): 4302–15.

Poggio, Laura, Luis M. de Sousa, Niels H. Batjes, Gerard B. M. Heuvelink, Bas Kempen, Eloi Ribeiro, and David Rossiter. 2021. “SoilGrids 2.0: Producing Soil Information for the Globe with Quantified Spatial Uncertainty.” *SOIL* 7 (1): 217–40.

Schielzeth, Holger, Niels J. Dingemanse, Shinichi Nakagawa, David F. Westneat, Hassen Allegue, Céline Teplitsky, Denis Réale, Ned A. Dochtermann, László Zsolt Garamszegi, and Yimen G. Araya-Ajoy. 2020. “Robustness of Linear Mixed-effects Models to Violations of Distributional Assumptions.” *Methods in Ecology and Evolution* 11 (9): 1141–52.
